# Disentangling genetic structure for genetic monitoring of complex populations

**DOI:** 10.1111/eva.12622

**Published:** 2018-03-23

**Authors:** Brook G. Milligan, Frederick I. Archer, Anne‐Laure Ferchaud, Brian K. Hand, Elizabeth M. Kierepka, Robin S. Waples

**Affiliations:** ^1^ Department of Biology New Mexico State University Las Cruces NM USA; ^2^ NOAA Fisheries Southwest Fisheries Science Center La Jolla CA USA; ^3^ Institut de Biologie Intégrative et des Systèmes (IBIS) Université Laval Québec QC Canada; ^4^ Flathead Lake Biological Station University of Montana Polson MT USA; ^5^ Biology Department Trent University Peterborough ON USA; ^6^ NOAA Fisheries Northwest Fisheries Science Center Seattle WA USA

**Keywords:** Λ‐coalescent, density, dispersal, genetic monitoring, isolation by distance, multiple merger coalescent, population structure, spatial Λ‐Fleming‐Viot model

## Abstract

Genetic monitoring estimates temporal changes in population parameters from molecular marker information. Most populations are complex in structure and change through time by expanding or contracting their geographic range, becoming fragmented or coalescing, or increasing or decreasing density. Traditional approaches to genetic monitoring rely on quantifying temporal shifts of specific population metrics—heterozygosity, numbers of alleles, effective population size—or measures of geographic differentiation such as *F*_ST_. However, the accuracy and precision of the results can be heavily influenced by the type of genetic marker used and how closely they adhere to analytical assumptions. Care must be taken to ensure that inferences reflect actual population processes rather than changing molecular techniques or incorrect assumptions of an underlying model of population structure. In many species of conservation concern, true population structure is unknown, or structure might shift over time. In these cases, metrics based on inappropriate assumptions of population structure may not provide quality information regarding the monitored population. Thus, we need an inference model that decouples the complex elements that define population structure from estimation of population parameters of interest and reveals, rather than assumes, fine details of population structure. Encompassing a broad range of possible population structures would enable comparable inferences across biological systems, even in the face of range expansion or contraction, fragmentation, or changes in density. Currently, the best candidate is the spatial Λ‐Fleming‐Viot (SLFV) model, a spatially explicit individually based coalescent model that allows independent inference of two of the most important elements of population structure: local population density and local dispersal. We support increased use of the SLFV model for genetic monitoring by highlighting its benefits over traditional approaches. We also discuss necessary future directions for model development to support large genomic datasets informing real‐world management and conservation issues.

… the development of statistical procedures to uncover the demographic or selection history of a set of populations that best explains the observed genetic structure is certainly one of the most interesting challenges of population genetics.

—L. Excoffier (2007)

## 1 TRADITIONAL GENETIC MONITORING

Genetic monitoring is concerned with estimating temporal changes in population demographic processes such as abundance, vital rates, and rates of exchange using information obtained from molecular markers (Schwartz, Luikart, & Waples, 2007). With the evolution of low‐cost, high‐throughput next‐generation sequencing methods, there is greater power to detect changes over time or space. This greatly facilitates discovery of population structure and makes genetic monitoring a valuable source of information for conservation policy decisions that would be difficult to obtain otherwise (Allendorf, England, Luikart, Ritchie, & Ryman, 2008; Duforet‐Frebourg & Blum, 2013; Fromentin, Ernande, Fablet, & de Pontual, 2009; Kardos, Taylor, Ellegren, Luikart, & Allendorf, 2016; Laikre et al., 2009; Lankau, Jørgensen, Harris, & Sih, 2011; Leblois et al., 2014; Lloyd, Campbell, & Neel, 2013; Mijangos, Pacioni, Spencer, & Craig, 2015; Ovenden, Berry, Welch, Buckworth, & Dichmont, 2015; Paz‐Vinas et al., 2013; Pierson et al., 2016; Rodrguez‐Trelles & Rodrguez, 2010; Waples, Punt, & Cope, 2008).

However, because studies can span long time frames and also incorporate results of other studies, care must be taken to ensure that inferences reflect actual population processes rather than changing molecular techniques (Allendorf, 2017; Charlesworth & Charlesworth, 2017) or incorrect model assumptions (Morin et al., 2010; Peery et al., 2012; Samarasin, Shuter, Wright, & Rodd, 2017). Moreover, populations tend to be complex in structure and change through time by expanding or contracting their geographic range, becoming fragmented or coalescing, or increasing or decreasing density (Hey & Machado, 2003). Indeed, all of these can be occurring simultaneously in different parts of a single species’ geographic range, and are more likely occurring in species of conservation concern (Whitlock & McCauley, 1999). While these changes are often in and of themselves important to conservation and basic population genetics, they can also cause challenges in the interpretation of analyses that are often overlooked.

In traditional approaches to genetic monitoring, the predominant approach quantifies patterns of variation or differentiation using measures such as heterozygosity, nucleotide diversity, numbers of alleles and percentage of polymorphic loci, and estimates of effective population size, *N*_*e*_ (Aravanopoulos, 2011; Excoffier, 2007; Schwartz et al., 2007; Tallmon et al., 2010). The underlying assumption is that temporal changes in these quantities are related to demographic parameters of conservation concern (Hoffmann & Willi, 2008; Pertoldi, Bijlsma, & Loeschcke, 2007; Schwartz et al., 2007). However, these relationships can be affected by changes in population processes (Schwartz et al., 2007) and by the number and type of genetic markers used and how closely they adhere to the analytical assumptions (Narum et al., 2008; Smith & Seeb, 2008; Smith et al., 2007). Consequently, metric‐based approaches to genetic monitoring or to quantifying population structure can be misleading when the necessary a priori assumptions are incorrect.

As an example, one of the most commonly used measures of differentiation is *F*_ST_, which was originally defined by Wright (1965) as the correlation of two alleles randomly sampled from a single subpopulation relative to the correlation of two alleles randomly sampled from the population as a whole. Under some conditions, *F*_ST_ is also related to the inverse of the migration rate: $F_{\mathrm{ST}}\approx 1/\left(4N_{e}m+1\right)$, where *N*_*e*_ *m* is the effective number of reproducing migrants per generation (Wright, 1931). This relationship has led to widespread use of *F*_ST_ as an indirect measure of gene flow (Slatkin, 1985).

However, this relationship is based on Wright's island model of population structuring in which all members of a population have an equal probability of contributing gametes to the next generation, generations are temporally nonoverlapping, all members of a population have an equal and constant probability of migrating, all populations are the same constant size, and populations are in equilibrium with respect to migration and genetic drift (Wright, 1931). While this model has proven to be a useful simplification, it is widely recognized that in most empirical populations these assumptions are practically never satisfied (Waples, 1998; Whitlock & McCauley, 1999). In fact, populations of conservation concern are very likely to demonstrate deviations from ideal conditions. These populations often change in size rapidly and are not in equilibrium (Archer et al., 2010; Whitlock & McCauley, 1999). A genetic monitoring study of such species that compares values of *F*_ST_ among samples from different time points, each of which can be out of equilibrium to differing degrees, is likely to be misleading, because estimates of gene flow derived from *F*_ST_ integrate long‐term demographic effects (Neigel, 2002). Strand, Milligan, and Pruitt (1996) also demonstrated that *F*_ST_ is informative about gene flow only if equilibrium under Wright's island model is assumed; while alternatively, the same value of *F*_ST_ is informative about the time since population divergence only if a strict radiation model of subdivision with no gene flow is assumed.

Finally, for most standard tests of population structure, there is a requirement that the samples are a priori partitioned into discrete populations. Population stratification schemes are necessary simplifications of real population structure and are often hypotheses being tested with the data at hand. Unless independent sources of data exist for comparison (Charpentier et al., 2012; Musiani et al., 2007), it can be difficult to assess how well putative stratifications reflect real populations. However, even when such datasets exist, population stratification defined by genetic data often differs from stratification defined by, for example, morphology or behavior, because they are influenced differently by demography and selection (Ortego, Garca‐Navas, Noguerales, & Cordero, 2015; Serrouya et al., 2012). In the absence of independent sources of data, populations are usually defined either based on how samples have been collected or as perceived centers of density within the species’ distribution, both of which can be biased by collection methods and might not reflect actual distribution or mating patterns.

Thus, most uses and interpretations of gene flow from estimates of *F*_ST_ are accompanied by implicit acceptance of a particular model of population structure, and their relevance depends crucially on the appropriateness of the model used to relate the pattern‐based quantities to underlying biological processes of interest. Further, models of population structure and models of population size change can make identical predictions for observable genetic quantities, and therefore, these processes cannot be distinguished without considering the full distribution of genetic variation (Mazet, Rodrguez, & Chikhi, 2015; Mazet, Rodríguez, Grusea, Boitard, & Chikhi, 2016). In the context of genetic monitoring, differentiating these is of crucial importance, so confounding them as a consequence of a priori assumptions is a serious issue. The inherent complexity of populations therefore poses a nontrivial problem for the prospect of discovering population structure, and presents significant challenges to the development of a coherent means of monitoring populations using genetic information gathered over any reasonably large spatiotemporal extent (Crandall, Bininda‐Emonds, Mace, & Wayne, 2000; Excoffier, 2007; Segelbacher et al., 2010). Nevertheless, this is a problem that must be addressed. What follows is our view of the path forward.

## 2 THEORY AND REALITY IN POPULATION GENETICS

**Figure 1:**
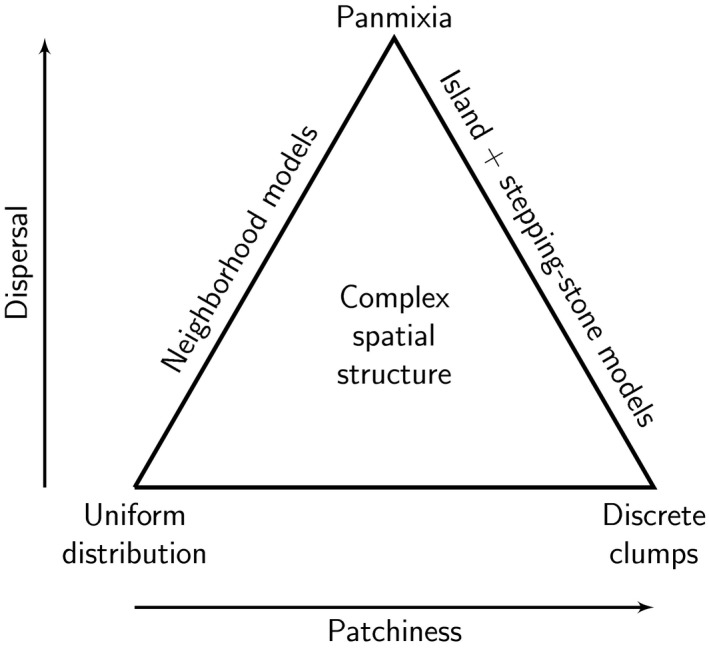
The parameter space for complex populations. Populations with complex spatial structure are located within a parameter space defined by dimensions corresponding to the degrees of patchiness and connectivity. For simplicity, an additional dimension corresponding to the local population density is not shown. Increasing connectivity for any population structure converges to the same outcome, that is, panmixia, so the feasible parameter space is shown as triangular

The rich theoretical foundation of population genetics has inspired numerous models to describe how genetic characteristics vary over space and time. This creates a challenge for discovering population structure or guiding genetic monitoring, because choices among models must be made a priori and available models might not correspond to biological reality. The range of patterns of structure in natural populations can be viewed as a triangular space described by patchiness and individual dispersal distance (Figure 1). If both patchiness and dispersal are low, individuals are relatively uniformly distributed. As patchiness increases, individuals become more clumped into discrete populations. As dispersal increases, all cases converge to a single panmictic population. In reality, groups of individuals within a metapopulation can exist at multiple locations in this space. Certainly for the discovery of population structure and often for the purposes of genetic monitoring, we are interested in where in this space a set of individuals lies, whether the location is shifting over time, and if so, the rate of change. To maximize analytical tractability, however, traditional population genetics models typically make simplifying assumptions about life histories and demographic and evolutionary processes. This limits their applicability by interpreting the study system with respect to a small subset of the parameter space.

In the most widely adopted paradigm, individuals are assumed to assort themselves into semi‐discrete subpopulations, within which matings occur at random. The two most commonly used models of this class are Wright's island model, introduced in Wright (1931) but not named until Wright (1943), and the stepping‐stone model (Kimura & Weiss, 1964; Weiss & Kimura, 1965). These models limit themselves to the right border of the spatial structure triangle (Figure 1). Here, subpopulations are convenient, and often necessary, units for subsequent analyses of genetic diversity within (heterozygosity, allelic and nucleotide diversity) and among (*F*_ST_ and related measures) groups of individuals. The primary parameters governing these models are the effective size of each subpopulation (*N*_*e*_) and the rate of migration among subpopulations (in the island model, *m* is the single migration rate among all subpopulations; in the stepping‐stone model, *m*_*j*_ is the migration rate among subpopulations separated by *j* steps and $m_{\infty }$ is the rate of long‐range migration, equivalent to *m* in the island model). Spatial heterogeneity is captured mainly through analysis of pairwise combinations of connected, discrete populations (Rousset, 1997; Slatkin, 1993), or by the estimation of migration matrices (Beerli & Felsenstein, 2001).

In contrast, the most widely adopted alternative paradigm is Wright's IBD model (Wright, 1943, 1946), which focuses on individuals assumed to be distributed continuously and uniformly across space. These models limit themselves to the left border of the spatial structure triangle (Figure 1). Here the primary parameters governing the models are local density (*d*) and the variance of parent–offspring dispersal distance ($\sigma^{2}$). Together these define the concept of neighborhood size as the geographic area within which most matings take place. Spatial heterogeneity is generally not considered in these models.

Some attempts to bridge these two paradigms have been made, but they are limited to identifying special cases that can transform one into the other. Stepping‐stone models, for example, converge to Wright's island model if migration rates except for $m_{\infty }$ are zero (Kimura & Weiss, 1964; Weiss & Kimura, 1965). Conversely, as the number of subpopulations increases and effective size of each becomes arbitrarily small, the stepping‐stone model approaches the IBD model. Kimura and Weiss (1964) suggested that their stepping‐stone model could be analyzed in terms of IBD by replacing *m*_1_ with $\sigma^{2}$ and by substituting the effective density *d*(*N*_*e*_ */N*) for *N*_*e*_.

Importantly, neither dominant paradigm penetrates the interior of the spatial structure parameter space (Figure 1), which creates problems when models based on those paradigms are used to discover population structure or are applied to genetic monitoring. Although some real‐world species fall neatly into one or the other of these paradigms, many others exist somewhere in the interior space of the triangle. In some species, individuals are neither randomly distributed across the landscape nor neatly clumped into semi‐discrete subpopulations, while for others individuals are arrayed in different spatial patterns in different areas and/or at different times. And for many other species, connectivity depends strongly on features of the habitat (which might change at different spatiotemporal scales) rather than being a simple function of distance as implied by the IBD model.

## 3 INDIVIDUALLY BASED LANDSCAPE GENETICS MODELS

In general, the area within the spatial structure triangle (Figure 1) can be considered the domain of landscape genetics, which integrates population genetics, landscape ecology, and spatial statistics to identify landscape and environmental factors that affect genetic and genomic variation (Milligan, 2017; Segelbacher et al., 2010). Landscape genetics, a term coined in 2003 (Manel, Schwartz, Luikart, & Taberlet, 2003) to describe increasingly spatially explicit advances in population genetics (Dyer, 2015a), has had a strong focus on the flow of genetic information across the landscape and hence population structure. Further, it is well recognized that model output and inference in landscape genetics is heavily influenced by and dependent on the scale and resolution (i.e., how finely resolved are measures of ecological differences) of ecological processes (e.g., dispersal and demography) that influence gene flow and population structure (Cushman & Landguth, 2010; Galpern & Manseau, 2013; Hand, Cushman, Landguth, & Lucotch, 2014; Wasserman, Cushman, Schwartz, & Wallin, 2010).

Most landscape genetic studies rely strongly on the dichotomy of individual versus population‐based models for inference (Dyer, 2015a; Storfer, Murphy, Spear, Holderegger, & Waits, 2010). The approach of using pattern‐based measures such as *F*_ST_ and correlating them with spatial and/or environmental factors, has long dominated landscape genetics (Waits & Storfer, 2016). These approaches require a priori stratification of samples into putative populations. Newer approaches like population graph approaches (Dyer, 2007, 2015b; Dyer & Nason, 2004; Murphy, Dyer, & Cushman, 2016) have been largely applied in population‐based frameworks, often where sampling locations, not genetically discrete populations, define the vertices of the graph. Individual‐based analyses in landscape genetics can help overcome problems with predefining populations, and many landscape genetic statistics can be adapted to individual‐based measures of genetic differentiation. However, individual‐based studies often yield thousands of pairwise values, making it difficult to make biologically relevant inferences of genetic structure (Kierepka & Latch, 2015). Furthermore, popular tests of association between matrices of pairwise distances, for example, Mantel tests, suffer from statistical errors (Graves, Beier, & Royle, 2012; Kierepka & Latch, 2015) and are easily susceptible to sampling biases (Kierepka & Latch, 2015; Oyler‐McCance, Fedy, & Landguth, 2013; Schwartz & McKelvey, 2009). Thus, despite its promise, much of the core of landscape genetics must be improved before it is ready to tackle the challenges of long‐term genetic monitoring and discovery of population structure.

Improvement of landscape genetics models for genetic monitoring might start from either of two points. The first is the family of spatially explicit, individually based ancestry clustering models, which includes geneland (Guillot, Estoup, Mortier, & Cosson, 2005), TESS (Chen, Durand, Forbes, & François, 2007), BAPS (Corander & Marttinen, 2006), and POPS (Jay, Durand, François, & Blum, 2015), many of which are derived from the nonspatial structure model (Falush, Stephens, & Pritchard, 2003; Pritchard, Stephens, & Donnelly, 2000). All of these models interpret the observed multilocus genotypes as samples from putative populations, which are inferred during the modeling process. As a consequence, they are limited to the right border of the spatial parameter space (Figure 1). In addition, a range of covariates are often included. For example, structure (Pritchard et al., 2000) allows prior distributions to be influenced by the sampled spatial location of each individual, while geneland (Guillot et al., 2005), TESS (Chen et al., 2007), spatial BAPS (Corander, Sirén, & Arjas, 2008), and POPS (Jay et al., 2015) explicitly include the sampled spatial location of each individual in the model. In addition, POPS (Jay et al., 2015) explicitly includes environmental as well as spatial information. However, none of these models explicitly includes gene flow, despite it being one of the most important genetic mechanisms influencing variability and local adaptation (Holderegger & Wagner, 2008). Thus, despite their promise, these models also need improvement if they are to be used to handle the complexities of long‐term genetic monitoring. Specific areas of improvement include the addition of more biologically relevant mechanisms such as gene flow in ways that acknowledge the spatial heterogeneity required for genetic monitoring and discovery of population structure (Milligan, 2017).

The second family contains the individually based explicitly genealogical models of ancestry, which are based upon the coalescent (Kingman, 1982). This includes a large set of models that infer, generally from DNA sequence data, such quantities as effective population size and growth rate, gene flow, and population divergence (Kuhner, 2008). Unlike most of the models in the first category, these are not truly spatially explicit; at best individuals are gathered into predefined populations for analysis using a structured coalescent (Hudson, 1990; Notohara, 1990). Furthermore, many of the parameters inferred in these models are averages across the entire sample. Thus, for example, spatially dependent density or gene flow cannot be ascertained, both of which are important for long‐term genetic monitoring or for discovery of population structure. As a result, while offering much promise, this set is likewise not immediately suitable.

The main approaches to population and landscape genetics provide strong foundations for genetic monitoring. However, they generally require making a priori assumptions about quantities that are the subject of inference and the models exhibit many problems when applied to the challenge of genetic monitoring (Table 1). Consequently, a new look at genetic monitoring and discovery of population structure is required.

**Table 1:** Current problems in the implementation of genetic monitoring models and important qualities of a genetic monitoring model

Primary problem	Examples of potential consequences	Improvements needed in genetic monitoring models
Current metrics heavily influenced by scale and vary greatly depending on the scale used	Multi‐scale studies show that landscape effects are evident at one scale and absent at another (Balkenhol et al., 2014; Millete & Keyghobadi, 2015)	Scale‐independent quantification of local population structure and connectivity
		Spatial heterogeneity in model parameters
Many genetic metric models require assignment of individuals to predetermined groups	Potential for erroneous groups from clustering algorithms (Frantz, Cellina, Krier, Schley, & Burke, 2009; Latch, Dharmarajan, Glaubitz, & Rhodes, 2006; Schwartz & McKelvey, 2009)	No a priori grouping
Genetic metrics are often divorced from the underlying genetic process, leading to poor estimation of the process itself	Inaccurate estimates of migration rates, especially at low values of *F*_ST_ (Allendorf, Luikart, & Aitken, 2013)	Directly incorporate known population genetics mechanisms
	Violation of assumptions can greatly impact estimates of effective population size (Neel et al., 2013)	
Genetic metrics can be sensitive to the marker type used and could therefore change temporally based solely on the methodology	Different spatial genetic structures between marker types (Bradbury et al., 2015)	Technology independent
	Limited applicability across studies for wide‐ranging species (de Groot et al., 2016)	

## 4 MODELS FOR GENETIC MONITORING AND DISCOVERY OF POPULATION STRUCTURE

A more general approach to population genetic analysis must place the focal system within the spatial structure triangle (Figure 1) as a natural outcome of the analysis, not start with a priori assumptions about its location within the parameter space. Additionally, the model would directly quantify the full distribution of actual population or evolutionary processes of interest as best as possible, decoupling these parameters from the elements that define population structure (Excoffier, 2007). In particular, this model would:

- Encompass a broad range of possible population structures, so that inferences made would be comparable across different geographic scales and types of biological systems,
- Utilize spatial information,
- Simultaneously quantify processes influencing population structure and connectivity, and assess changes in both over time,
- Allow for spatial heterogeneity in model parameters,
- Directly estimate parameters of interest and their uncertainty, while not being confounded by range expansion or contraction, fragmentation, or changes in density, and
- Be compatible with multiple types of genetic data, allowing it to be informed by legacy microsatellite or potentially allozyme data sets, next‐generation sequencing data, or data generated by future technologies.

The basic observations for a general analysis with this hypothetical model would be multilocus genotypes, multilocus sequences, or full genome sequences of individuals, their geographic locations, and information on covariates that might influence local density, movement, and selection. The model should serve as a bridge between the two main paradigms of individual neighborhood and island/stepping‐stone models (i.e., the left and right borders of the spatial structure triangle (Figure 1)), and encompass these models as boundary conditions. Preliminary analyses using the model might indicate that a given system fits comfortably onto either border, justifying the use of one or the other set of standard analytical regimes. However, most empirical cases are more likely to lie in the interior, so the model could also give an indication of the appropriateness of measures deriving from one or the other of the main paradigms.

## 5 SPATIAL Λ‐FLEMING‐VIOT MODEL

Currently, the only model with immediate potential to address most of the requirements for long‐term genetic monitoring is the spatial Λ‐Fleming‐Viot (SLFV) model (Barton, Etheridge, & Véber, 2013; Guindon, Guo, & Welch, 2016; Joseph, Hickerson, & Alvarado‐Serrano, 2016; Kelleher, Barton, & Etheridge, 2013). The SLFV is a spatially explicit extension of the Λ‐Fleming–Viot model which is itself an extension of the Fleming–Viot model (Fleming & Viot, 1979). Equivalently, it is a spatially explicit version of the Λ‐coalescent which is an extension of Kingman's coalescent (Kingman, 1982; Tellier & Lemaire, 2014). Specifically, coalescence in the SLFV model is not limited to two lineages, and individuals can be distributed arbitrarily across space, avoiding the restriction in classical island and stepping‐stone models of discrete population boundaries. As a result, the SLFV model permits the simultaneous, yet independent, estimation of local population density and local dispersal rates, two key parameters of population processes integral to genetic monitoring studies. The mathematical background for the SLFV model was introduced in Etheridge (2008) and is well described in Barton, Etheridge, and Véber (2010), Barton et al. (2013), Berestycki, Etheridge, and Véber (2013), and Véber and Wakolbinger (2015). Extensions to the model including selection, mutation, recombination, and skewed reproductive success are thoroughly covered by Dawson and Greven (2014), Etheridge and Véber (2012), Etheridge, Freeman, and Straulino (2017), and Montano (2016). Efficient implementations of the selectively neutral, spatially homogeneous SLFV model, with and without recombination, are described in Kelleher et al. (2013), Kelleher, Etheridge, and Barton (2014) and Kelleher, Etheridge, and McVean (2016). In what follows, we introduce informally this simple model, then present the steps involved in a more mathematically rigorous form to illustrate explicitly how the restrictive assumptions can be relaxed to obtain a model with the desired characteristics outlined in the previous section.

**Figure 2:**
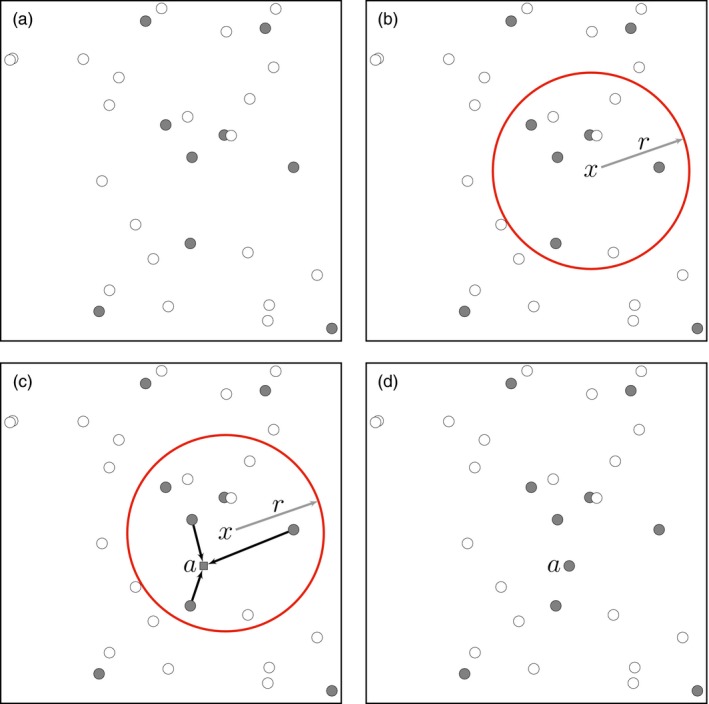
Illustration of one iteration of the SLFV model. (a) Initial condition involving individuals at their empirical sampling locations with two haplotypes (white and gray), (b) placement of a random neighborhood (circle) defined by its center (*x*) and radius (*r*), (c) random placement of a putative ancestor (square) and coalescence of ancestry of randomly selected descendants, and (d) distribution of remaining individuals after removal of the descendants

In its simplest form, the SLFV model constructs coalescent genealogies of subgroups of haploid individuals through iterations of reproduction and movement events backwards in time (Figure 2). The sequence begins with a set of individuals, arbitrarily distributed across a continuous landscape (Figure 2a), each carrying their empirical genotypic data (although they can also optionally be associated with other data such as sex, demographic or reproductive state). In the first step, a neighborhood center (*x*) and radius (*r*) are randomly selected (Figure 2b). All coalescent events will be limited to individuals within this neighborhood. A new location within the neighborhood is randomly selected for the ancestor (*a*) and its genotype is selected from the distribution in the neighborhood associated with that location (Figure 2c). Existing individuals within the neighborhood are then randomly selected to be descendants of the new ancestor. Finally, as for the Moran (1958) model, the descendants are removed, having been replaced by the ancestor (Figure 2d), and a new iteration begins, with iterations continuing until only a single ancestor remains.

As outlined below, the individuals need not be haploid. Sexual reproduction can be accommodated by selecting more than a single ancestor. Note that small‐scale, for example, single generation, reproduction events will necessarily involve two ancestors, but large‐scale events, that is, those with long intervals or covering large areas, can involve more than two because multiple generations might have intervened (Kelleher et al., 2013).

The steps in this process can be formalized to illustrate the generalizations that are possible. For clarity of exposition we will consider the single locus model, because it captures the spatially explicit nature that is crucial for genetic monitoring; multilocus extensions are straightforward (Kelleher et al., 2013, 2014, 2016). Consider a sample of *n*, not necessarily haploid, individuals, each from a known location *x* within a *d*‐dimensional landscape *L* and with a known state *s* (e.g., genotype, sex, etc.). Thus, each individual *i* can be represented by the quantities *i*,* x*_*i*_, and *s*_*i*_. Let *C*(*t*) be the set of individuals extant at time *t*; this can change at discrete points in time as reproductive or movement events occur. Initially, $C=\{\left(i,x_{i},\;s_{i}\right)\forall i\}$. Iterate through the following steps until *C* contains only a single individual, the ancestor of the entire sample.

1. Generate an event at a location, which will involve a mixture of reproduction and movement. To do so, sample a spatial probability distribution *E*(*x*) from a family of spatial distributions across the landscape *L*. In the simplest case (Kelleher et al., 2013), the family of distributions *E*(*x*) for a *d* + 1 dimensional landscape *L* is composed of uniform distributions within *d*‐spheres of radius *r* centered at points *e*. Alternatively, a Gaussian distribution for the selection has been used (Guindon et al., 2016). Nonhomogeneity in the landscape can be incorporated with different families of *E*(*x*), which might, for example, depend on the distribution of habitats, land use patterns, other environmental characteristics, or the state (genetic or demographic) of the individuals.
2. Select a set $C^{\prime }$ of individuals based upon the spatial distribution *E*(*x*). For every individual *j* in *C*, select it with a probability of $E(x_{j},s_{j})$. This will yield a set $C^{\prime }$ containing zero or more individuals, randomly selected according to the spatial distribution associated with the event and their state. In the case of no mutation, all individuals in $C^{\prime }$ will have the same state, but this restriction is not necessary. Depending on the number of individuals in $C^{\prime }$, this event either has no effect or involves a mixture of reproduction and movement.
  (a) If $C^{\prime }$ is empty, no individuals are affected by the event and *C* is unchanged. Construct a new event.
  (b) If $C^{\prime }$ contains at least one individual, the event is potentially a mixture of reproduction and movement (and possibly mutation). Sample a set of individuals, which will replace those in $C^{\prime }$, from the distribution $R(x|C^{\prime })$. Some or all of these individuals may be ancestors of (some of) those in $C^{\prime }$; the remainder are individuals in $C^{\prime }$ that have simply moved. Thus, the distribution $R(x|C^{\prime })$ determines the mixture of reproduction and movement that occurs in the event. For sexual reproduction, $R(x|C^{\prime })$ can generate locations for more than one ancestor, and even for more than two in the case of large‐scale events. In this case, ancestry must be distributed across the selected individuals; Kelleher et al. (2016) compares the efficiency of alternative algorithms for accomplishing this. In the simplest cases, $R(x|C^{\prime })$ is uniform across the *d*‐sphere defined by *E*(*x*) (Kelleher et al., 2013) or may only depend on the distance between individuals (Guindon et al., 2016). However, more complex distributions can depend on the locations of individuals in $C^{\prime }$, on environmental characteristics across *L*, or on individual states. If mutation is possible, sample the state of these replacement individuals from the distribution $S(s|C^{\prime })$. Finally, remove all individuals in $C^{\prime }$ from *C* and replace them with the newly sampled ones.

Clearly the SLFV model is very general. It is applicable to 1‐ or 2‐dimensional habitats, and the landscape can be homogeneous or heterogeneous in any way. The suitable locations for individuals can be continuously distributed (either uniformly or not) across the landscape, can be patchily distributed, can be limited to discrete positions, or can be a complex mixture of these. The flexibility of the SLFV model enables the spatial structure to emerge from the analysis rather than be imposed a priori. Developing software that reflects the range of applicability of the SLFV model remains an open challenge that is crucial to the advancement of genetic monitoring as well as population genetics.

The selectively neutral, spatially homogeneous SLFV model is dependent on several parameters, the two most important of which govern how $R(x|C^{\prime })$, the spatial distribution of new ancestors and coalescent events, reflects local population density and local dispersal rate. This means that the SLFV model is directly based on biological processes of known importance to the genetic composition of populations, a feature critical for genetic monitoring and discovery of population structure. For example, it explicitly models the processes of reproduction and local movement (Figure 2c), permitting direct inference of the spatial distribution of relevant population processes. This is in contrast to summary pattern‐based measures such as *F*_ST_ that can be related to biological mechanisms such as gene flow only if a population fits a particular model.

The data required for the SLFV model are those already generally obtained for genetic monitoring: individual‐specific genetic data, either multilocus genotypes or DNA sequences, and individual‐specific geographic locations. Additionally, spatially or temporally heterogeneous versions of the model could use spatial or temporal covariates, such as habitat characteristics, to parameterize the local population density and dispersal parameters. Analogous parameterizations are central to the success of landscape genetics (Balkenhol, Cushman, Storfer, & Waits, 2016; Manel et al., 2003), which seeks to relate landscape or environmental characteristics to, for example, dispersal through surfaces that quantify flow of individuals through the landscape (McRae, 2006).

Two applications of the SLFV model illustrate both its power and the importance of relaxing the assumptions incorporated into existing software. Joseph et al. (2016) developed an approximate Bayesian computation (ABC) pipeline based upon the selectively neutral, spatially homogeneous SLFV model (Kelleher et al., 2013, 2014). The pipeline was used to validate the estimation of neighborhood size from simulated data and subsequently to estimate both neighborhood size and dispersal radius from empirical data on *Berkheya cuneata* (Asteraceae) from South Africa. In their model, dispersal radius *R* was the maximum distance individuals could disperse, and neighborhood size was the number of individuals within the area of an event of radius *R*. For validation, 100,000 datasets were generated for eight individuals sampled at 10 unlinked loci. Each dataset was composed of the genealogy generated by the SLFV model and 1 kb sequences simulated along each genealogy. Data generation took 2 days on a 12‐core computer. Subsequently, the posterior distribution of neighborhood size was calculated using ABC based upon 100 replicate leave‐one‐out cross‐validations; regression of the estimated neighborhood size on the actual neighborhood size had *R* ^2^ = 0.87.

The empirical analysis of *Berkheya cuneata* used a total of 33 individuals with known locations and sequence data at one nuclear and two plastid loci (Joseph et al., 2016). The same pipeline implementing the selectively neutral, spatially homogeneous SLFV model was used to generate 100,472 datasets; rejection ABC was used to sample from the posterior distributions of both neighborhood size and dispersal distance. The median estimates of neighborhood size and dispersal distance were 502.50 (95% HPDI 56.03–962.00) and 7.33 km (HPDI 2.44–9.86 km), respectively. The process of generating datasets took 36 days to complete.

This study illustrates several important points regarding practical use of the SLFV model. First, the two most biologically important parameters, neighborhood size and dispersal distance, are identifiable; that is, they can be estimated separately using the SLFV model. Second, it is possible to obtain useful estimates even from relatively small datasets composed of no more than dozens of individuals or handfuls of loci. Third, there is room for improved computational efficiency to accommodate larger datasets. Finally, adding spatial heterogeneity in the form of known resistance surfaces or the like, as is often done in landscape genetics (McRae, 2006; Spear, Cushman, & McRae, 2016), will increase realism without adding parameters; inferring properties of resistance surfaces adds no more parameters than the equivalent multivariate regression or similar landscape genetic analysis would. Thus, while the existing pipeline (Kelleher et al., 2013, 2014) does not accommodate that flexibility, a spatially heterogeneous SLFV model is both feasible and likely to be computationally tractable.

A second example using the selectively neutral, spatially homogeneous SLFV model reinforces these points and illustrates additional ones. Guindon et al. (2016) also validated the SLFV model with simulations and applied it to data, in this case from influenza A virus (H1N1 subtype) for the flu seasons from 2009 to 2014. Instead of using ABC as did Joseph et al. (2016), Guindon et al. (2016) generated samples from the posterior distributions of the parameters with the Metropolis‐Hastings MCMC algorithm. For validation, 300 simulated datasets of 5,000 individuals were generated using the SLFV model to generate genealogies and the Kimura 2‐parameter model (Kimura, 1980) to generate nucleotide sequences given the genealogies. Effective population density (*d*) and dispersal intensity ($\sigma^{2}$) (Wright, 1946) were estimated using the SLFV model based upon a sample of 50 individuals sampled at either two or ten different sites. Additionally, parameter estimates were obtained using the structured coalescent (Hudson, 1990; Notohara, 1990) under the assumption of either two or ten discrete populations. Estimates from the structured coalescent were upwardly biased to a large degree, though much less so for ten than for two populations. Estimates from the SLFV model were much better, although the precision declined with larger values of dispersal intensity. These computations took 100 hr to complete on a computer with 2.7–2.8 GHz CPUs.

The empirical analysis of influenza (Guindon et al., 2016) was based upon two biological replicates, each involving one sequence of the NA segment of the influenza A virus (H1N1 subtype) per 48 contiguous state of the U.S.A. from each of the five flu seasons from 2009 to 2014. Each dataset yielded an estimate of the posterior distributions for neighborhood size $N_{s}\propto \sigma^{2}d$ and dispersal radius σ (Wright, 1946). Comparison of the five distributions for these two parameters revealed that the two biological replicates yielded similar distributions, an indication of consistency despite moderate sample size. Further, the 2009–2010 flu season was different from the other four; it was characterized by a smaller neighborhood size and a larger dispersal radius. This observation indicates limited infection rates and broader climatic tolerance, which is consistent with the known history (longer duration and milder incidence) of that epidemic.

This study reinforces the point that neighborhood size and dispersal rates can be estimated separately using the SLFV model. Distinguishing between them is important, especially in the case of genetic monitoring where either or both might shift (as they did with influenza) through time. Detecting those shifts may in fact be a major reason for undertaking a monitoring program. It also reinforces the point that useful estimates can be obtained for typical samples using a reasonable amount of computation. Thus, the SLFV model can be developed into a practical approach to genetic monitoring. It may also serve the task much better than other methods, such as those based upon *F*_ST_ or the structured coalescent, that impose a priori assumptions upon the spatial structure of the populations under study.

Although analyses using the SLFV model to date (Guindon et al., 2016; Joseph et al., 2016) have assumed spatial homogeneity in both neighborhood size and dispersal, there is no inherent reason not to allow spatial heterogeneity, just as it is routinely included in landscape genetics analysis (Balkenhol et al., 2016). For example, given information on the spatial layout of distinct habitat types, one could estimate different densities or dispersal rates for each habitat. In turn, those parameters could be the focus of genetic monitoring to detect changes in habitat‐specific density or dispersal, information that would be of great value to a monitoring program. It would also reveal valuable information on the basic biology of the species under study. Importantly, differences among habitats (or other spatially defined factors) would emerge naturally from the analysis if they exist rather than be imposed at the outset by selection of the analysis framework. Of course, as with landscape genetics models, SLFV models with too many parameters will be impossible to estimate. How many and which parameters can be estimated remains an open question, and software implementations of more complex, and possibly biologically realistic, models are required to investigate this.

## 6 POTENTIAL SHORTCOMINGS OF CURRENT IMPLEMENTATIONS OF THE SLFV MODEL

Current implementations of the SLFV model (Guindon et al., 2016; Kelleher et al., 2013, 2016) include restriction to selectively neutral markers and spatially homogeneous landscapes. Inefficiencies of implementation or limited sets of MCMC operators might also be shortcomings leading to analyses taking longer to complete or being limited in scope. These are purely technical limitations related to the early stage of development of the SLFV model, and can be overcome by improvements in software design coupled with additional investigation of model performance. Given that coalescent models have recently been extended to genome‐scale data for phylogenetic analysis (Bansal, Burleigh, & Eulenstein, 2010; Boussau et al., 2013; Jenkins, Fearnhead, & Song, 2015; Kumar, Hallström, & Janke, 2013), it is likely that the same will be true for the SLFV model.

A feature of the SLFV model as currently implemented is that no distinction, other than location, is made among individuals with respect to their likelihood of birth; in the backward in time version of the model described above, the probability distribution *E*(*x*) that selects individuals influenced by an event depends only on location. Greater biological realism could be incorporated into the model by allowing *E*(*x*) to depend on, for example, the demographic state of individuals or their genotype. These states need not even be static; they could be projected through time from one event to the next much as phylogenetic analysis projects state change along lineages. Further, these projections could incorporate structured population models (Caswell, 2000) in a natural way.

Like the Moran (1958) model, the SLFV model applies to overlapping generations, as reproductive events are not synchronized across the population in any way other than by the geographic scale of each event. Interestingly, this feature contrasts with most other models, which have the opposite limitation of applying to nonoverlapping generations. As many biological life cycles involve overlapping generations, this gives the SLFV model greater practical relevance than discrete generation models.

Despite these limitations of implementation, the SLFV model is already useful for separate estimation of such biologically meaningful parameters as local population density and dispersal, which are confounded in other models. Current software implementations assume that individuals are distributed uniformly in space, so variation in density must be discovered by modeling different spatial partitions. However, as outlined above this is a technical limitation of the current implementations not of the SLFV model itself. One priority, therefore, is to generalize the implementations to match the potential of the model so that population structure need not be imposed in advance but can be obtained as a direct outcome of analysis. This would enable discovery of the nature of populations or monitoring their state over time or space in ways that are impossible if the structure of the populations must be assumed a priori. For this reason, the SLFV model offers distinct advantages both for the advancement of our understanding of population genetics and our application of it to genetic monitoring.

## 7 A LONG‐TERM GENETIC MONITORING STRATEGY

What would a long‐term genetic monitoring strategy based upon spatially explicit coalescent models, such as the spatial Λ‐Fleming‐Viot model, look like? From the data acquisition viewpoint, such a monitoring strategy would largely resemble any other. Geo‐referenced samples of individuals would be distributed across the species range, and sampling would be repeated to create a time series. Environmental and landscape data would be obtained as well to provide information on potential covariates. As with all similar studies, the goal of sampling is to ensure that each individual is equally likely to be sampled, that individuals are sampled independently, and that the environmental and landscape covariates are spatially representative.

From the data analysis viewpoint, however, such a monitoring strategy would look quite different from common practice. First, different types of genetic data, for example, DNA sequences and multilocus genotypes would be analyzed simultaneously in the same model. In principle, this has long been possible for coalescent‐based methods (Beerli & Palczewski, 2010; Bouckaert et al., 2014; Drummond & Rambaut, 2007); however, in practice different types of data, for example, single nucleotide polymorphisms (SNPs) and microsatellites, are analyzed separately. For genetic monitoring, the focus is on basic properties of the populations, for example, spatially dependent density and dispersal, not on data type‐specific estimates (Milligan, Leebens‐Mack, & Strand, 1994). Joint analysis of the data is likely to be better than independent analyses of partitions, in much the same way that joint analysis of gene trees leads to better inference of species trees in phylogenetics (Liu, Xi, Wu, Davis, & Edwards, 2015).

Second, increasing emphasis would be placed on the posterior distributions of parameters, as opposed to their point estimates. Much as Guindon et al. (2016) were able to recognize similarities and differences among distributions inferred for a sequence of influenza outbreaks, genetic monitoring must recognize similarities and differences in parameters across spatial and temporal dimensions. This can only be done accurately if information on the full distributions is available.

Third, the same model would be used for temporal comparisons to identify biological, not methodological, shifts. Not only would this make comparisons more meaningful, it would also enable direct and quantitative analysis of changes. The current practice of using different data and models over time, coupled with ad hoc interpretations of the differences, does not lend itself to reliable monitoring protocols.

Finally, the nature of the models used must of course be improved so that they will handle these demands. They must cover a full range of data types and include a full range of biological mechanisms to achieve this. Consequently, advances in genetic monitoring depend crucially on advances in the models and analyses that are possible. The rapid technological advances in data acquisition, for example, the increasing accessibility of genome‐scale data, make it easy to forget that the data are meaningless without suitable analyses. For long‐term genetic monitoring, those analyses must yield comparable information, and they must do so in the face of both dynamically changing populations and changing types of data.

## 8 CONCLUSIONS

In conservation biology, there has been a movement toward better utilizing genomic data and information about adaptive genetic markers to improve our understanding of evolutionary processes, rates of dispersal, local adaptation, genotype‐by‐environment interactions, and other important factors influencing population structure at multiple scales (Allendorf, Hohenlohe, & Luikart, 2010; Garner et al., 2016). By enabling process‐based, rather than pattern‐based, approaches, models such as the spatial Λ‐Fleming‐Viot model will allow the quantitative, spatiotemporal comparisons required for rigorous and informative genetic monitoring and for discovering the structure of natural populations. They will also allow adaptive incorporation of additional monitoring effort to efficiently reduce uncertainties and iteratively improve inferences about temporal changes in monitored systems. Finally, they will allow integration of new samples, including historical ones from archival collections, into a monitoring effort, thereby greatly expanding the time scale over which monitoring can meaningfully occur. As a consequence of the parallel development of these models and genetics technology, genetic monitoring stands poised to provide a rich source of information for more effectively guiding real‐time management decisions, monitoring the impact of human activities including changes in policy, and informing us about fundamental biological processes such as responses to global climate change.

## ACKNOWLEDGEMENTS

This work was assisted through participation in the Next Generation Genetic Monitoring Investigative Workshop at the National Institute for Mathematical and Biological Synthesis and sponsored by the National Science Foundation through NSF Award #DBI‐1300426, with additional support from The University of Tennessee, Knoxville. Any opinions, findings, and conclusions or recommendations expressed in this material are those of the authors and do not necessarily reflect the views of the National Science Foundation. BKH was partially supported by funds from NSF (award #DOB‐1639014) and NASA (award #NNX14AB84G). We thank two anonymous reviewers for comments that greatly improved our writing.

## DATA ARCHIVING STATEMENT

There are no data associated with this article.

## CONFLICT OF INTEREST

None declared.

## References

[eva12622-bib-0001] Allendorf, F. W. (2017). Genetics and the conservation of natural populations: Allozymes to genomes. Molecular Ecology, 26, 420–430. 27933683 10.1111/mec.13948

[eva12622-bib-0002] Allendorf, F. W. , England, P. R. , Luikart, G. , Ritchie, P. A. , & Ryman, N. (2008). Genetic effects of harvest on wild animal populations. Trends in Ecology and Evolution, 23, 327–337. 18439706 10.1016/j.tree.2008.02.008

[eva12622-bib-0003] Allendorf, F. , Hohenlohe, P. , & Luikart, G. (2010). Genomics and the future of conservation genetics. Nature Reviews Genetics, 11, 697–709. 10.1038/nrg2844 20847747

[eva12622-bib-0004] Allendorf, F. W. , Luikart, G. , & Aitken, S. N. (2013). Conservation and the genetics of populations, 2nd ed Hoboken, New Jersey: Wiley‐Blackwell.

[eva12622-bib-0005] Aravanopoulos, F. A. (2011). Genetic monitoring in natural perennial plant populations. Botany‐Botanique, 89, 75–81.

[eva12622-bib-0006] Archer, F. I. , Martien, K. K. , Taylor, B. L. , LeDuc, R. G. , Ripley, B. J. , Givens, G. H. , & George, J. C. (2010). A simulation‐based approach to evaluating population structure in non‐equilibrial populations. Journal of Cetacean Research and Management, 11, 101–113.

[eva12622-bib-0007] Balkenhol N., Cushman S. A., Storfer A. T., & Waits L. P. (Eds.) (2016). Landscape genetics: Concepts, methods, applications. Hoboken, New Jersey: Wiley Blackwell.

[eva12622-bib-0008] Balkenhol, N. , Holbrook, J. D. , Onorato, D. , Zager, P. , White, C. , & Waits, L. P. (2014). A multi‐method approach for analyzing hierarchical genetic structures: A case study with cougars *Puma concolor* . Ecography, 37, 1–12.

[eva12622-bib-0009] Bansal, M. S. , Burleigh, J. G. , & Eulenstein, O. (2010). Efficient genome‐scale phylogenetic analysis under the duplication‐loss and deep coalescence cost models. BMC Bioinformatics, 11(supplement 1), S42 10.1186/1471-2105-11-S1-S42 20122216 PMC3009515

[eva12622-bib-0010] Barton, N. H. , Etheridge, A. M. , & Véber, A. (2010). A new model for evolution in a spatial continuum. Electronic Journal of Probability, 15(7), 162–216.

[eva12622-bib-0011] Barton, N. H. , Etheridge, A. M. , & Véber, A. (2013). Modelling evolution in a spatial continuum. Journal of Statistical Mechanics: Theory and Experiment, 2013(01), P01002 10.1088/1742-5468/2013/01/P01002

[eva12622-bib-0012] Beerli, P. , & Felsenstein, J. (2001). Maximum likelihood estimation of a migration matrix and effective population sizes in *n* subpopulations by using a coalescent approach. Proceedings of the National Academy of Science, 98, 4563–4568. 10.1073/pnas.081068098 PMC31874 11287657

[eva12622-bib-0013] Beerli, P. , & Palczewski, M. (2010). Unified framework to evaluate panmixia and migration direction among multiple sampling locations. Genetics, 185, 313–326. 20176979 10.1534/genetics.109.112532 PMC2870966

[eva12622-bib-0014] Berestycki, N. , Etheridge, A. M. , & Véber, A. (2013). Large scale behaviour of the spatial Λ‐Fleming‐Viot process. Annales de l'Institut Henri Poincaré, Probabilités et Statistiques, 49, 374–401.

[eva12622-bib-0015] Bouckaert, R. , Heled, J. , Kühnert, D. , Vaughan, T. , Wu, C.‐H. , Xie, D. , … Drummond, A. J. (2014). BEAST 2: A software platform for Bayesian evolutionary analysis. Computational Biology, 10(4), e1003537 10.1371/journal.pcbi.1003537 24722319 PMC3985171

[eva12622-bib-0016] Boussau, B. , Szöllosi, G. J. , Duret, L. , Gouy, M. , Tannier, E. , & Daubin, V. (2013). Genome‐scale coestimation of species and gene trees. Genome Research, 23, 323–330. 23132911 10.1101/gr.141978.112 PMC3561873

[eva12622-bib-0017] Bradbury, I. R. , Hamilton, L. C. , Dempson, B. , Robertson, M. J. , Bourret, V. , Bernatchez, L. , & Verspoor, E. (2015). Transatlantic secondary contact in Atlantic salmon, comparing microsatellites, a single nucleotide polymorphism array, and restriction‐site associated DNA sequencing for the resolution of complex spatial structure. Molecular Ecology, 24, 5130–5144. 26407171 10.1111/mec.13395

[eva12622-bib-0018] Caswell, H. (2000). Matrix population models: Construction, analysis, and interpretation (2nd edn). Sunderland, MA: Sinauer.

[eva12622-bib-0019] Charlesworth, B. , & Charlesworth, D. (2017). Population genetics from 1966 to 2016. Heredity, 118, 2–9. 27460498 10.1038/hdy.2016.55 PMC5176116

[eva12622-bib-0020] Charpentier, M. J. E. , Fontaine, M. C. , Cherel, E. , Renoult, J. P. , Jenkins, T. , Benoit, L. , … Tung, J. (2012). Genetic structure in a dynamic baboon hybrid zone corroborates behavioural observations in a hybrid population. Molecular Ecology, 21, 715–731. 21988698 10.1111/j.1365-294X.2011.05302.x

[eva12622-bib-0021] Chen, C. , Durand, E. , Forbes, F. , & François, O. (2007). Bayesian clustering algorithms ascertaining spatial population structure: A new computer program and a comparison study. Molecular Ecology Notes, 7, 747–756.

[eva12622-bib-0022] Corander, J. , & Marttinen, P. (2006). Bayesian identification of admixture events using multilocus molecular markers. Molecular Ecology, 15, 2833–2843. 10.1111/j.1365-294X.2006.02994.x 16911204

[eva12622-bib-0023] Corander, J. , Sirén, J. , & Arjas, E. (2008). Spatial modelling of genetic population structure. Computational Statistics, 23, 111–129.

[eva12622-bib-0024] Crandall, K. A. , Bininda‐Emonds, O. R. P. , Mace, G. M. , & Wayne, R. K. (2000). Considering evolutionary processes in conservation biology. Trends in Ecology and Evolution, 15, 290–295. 10856956 10.1016/s0169-5347(00)01876-0

[eva12622-bib-0025] Cushman, S. A. , & Landguth, E. L. (2010). Scale dependent inference in landscape genetics. Landscape Ecology, 25, 967–979. 10.1111/j.1365-294X.2010.04656.x 20618896

[eva12622-bib-0026] Dawson, D. A. , & Greven, A. (2014). Spatial Fleming‐Viot models with selection and mutation. Lecture Notes in Mathematics: Springer.

[eva12622-bib-0027] Drummond, A. J. , & Rambaut, A. (2007). BEAST: Bayesian evolutionary analysis by sampling trees. BMC Evolutionary Biology, 7, 214 10.1186/1471-2148-7-214 17996036 PMC2247476

[eva12622-bib-0028] Duforet‐Frebourg, N. , & Blum, M. G. B. (2013). Nonstationary patterns of isolation‐by‐distance: Inferring measures of local genetic differentiation with Bayesian kriging. Evolution, 68, 1110–1123. 10.1111/evo.12342 PMC4285919 24372175

[eva12622-bib-0029] Dyer, R. J. (2007). The evolution of genetic topologies. Theoretical Population Biology, 71, 71–79. 16919694 10.1016/j.tpb.2006.07.001

[eva12622-bib-0030] Dyer, R. (2015a). Is there such a thing as landscape genetics? Molecular Ecology, 24, 3518–3528. 26011166 10.1111/mec.13249

[eva12622-bib-0031] Dyer, R. (2015b). Population graphs and landscape genetics. Annual Review of Ecology and Systematics, 46, 327–342.

[eva12622-bib-0032] Dyer, R. J. , & Nason, J. D. (2004). Population graphs: The graph theoretic shape of genetic structure. Molecular Ecology, 13, 1713–1727. 15189198 10.1111/j.1365-294X.2004.02177.x

[eva12622-bib-0033] Etheridge, A. M. (2008). Drift, draft and structure: Some mathematical models of evolution. Stochastic Models in Biological Sciences, 80, 121–144.

[eva12622-bib-0034] Etheridge, A. , Freeman, N. , & Straulino, D. (2017). The Brownian net and selection in the spatial Λ‐Fleming‐Viot process. Electronic Journal of Probability, 22(39), 1–36. 10.1214/17-EJP61

[eva12622-bib-0035] Etheridge, A. M. , & Véber, A. (2012). The spatial Λ‐Fleming‐Viot process on a large torus: Genealogies in the presence of recombination. Annals of Applied Probability, 22, 2165–2209. 10.1214/12-AAP842

[eva12622-bib-0036] Excoffier, L. (2007). Analysis of population subdivision In Balding D. J., Bishop M. & Cannings C. (Eds.), Handbook of statistical genetics (third, Chap. 29, pp. 980–1020). Chichester: Wiley.

[eva12622-bib-0037] Falush, D. , Stephens, M. , & Pritchard, J. K. (2003). Inference of population structure using multilocus genotype data: Linked loci and correlated allele frequencies. Genetics, 164, 1567–1587. 12930761 10.1093/genetics/164.4.1567 PMC1462648

[eva12622-bib-0038] Fleming, W. H. , & Viot, M. (1979). Some measure‐valued Markov processes in population genetics theory. Indiana University Mathematics Journal, 28, 817–843.

[eva12622-bib-0039] Frantz, A. C. , Cellina, S. , Krier, A. , Schley, L. , & Burke, T. (2009). Using spatial Bayesian methods to determine the genetic structure of a continuously distributed population: Clusters or isolation by distance? Journal of Applied Ecology, 46, 493–505.

[eva12622-bib-0040] Fromentin, J.‐M. , Ernande, B. , Fablet, R. , & de Pontual, H. (2009). Importance and future of individual markers for the ecosystem approach to fisheries. Aquatic Living Resources, 22, 395–408.

[eva12622-bib-0041] Galpern, P. , & Manseau, M. (2013). Finding the functional grain: Comparing methods for scaling resistance surfaces. Landscape Ecology, 28, 1269–1281. 10.1007/s10980-013-9873-1

[eva12622-bib-0042] Garner, B. A. , Hand, B. K. , Amish, S. J. , Bernatchez, L. , Foster, J. T. , Miller, K. M. , … Luikart, G. (2016). Genomics in conservation: Case studies and bridging the gap between data and application. Trends in Ecology and Evolution, 31, 81–83. 26654124 10.1016/j.tree.2015.10.009

[eva12622-bib-0043] Graves, T. A. , Beier, P. , & Royle, J. A. (2012). Current approaches using genetic distances produce poor estimates of landscape resistance to interindividual dispersal. Molecular Ecology, 22, 3888–3903. 10.1111/mec.12348 23786212

[eva12622-bib-0044] de Groot, G. A. , Nowak, C. , Skrbinek, T. , Andersen, L. W. , Aspi, J. , Fumagalli, L. , … Muñoz‐Fuentes, V. (2016). Decades of population genetic research reveal the need for harmonization of molecular markers: The grey wolf *Canis lupus* as a case study. Mammal Review, 46, 44–59.

[eva12622-bib-0045] Guillot, G. , Estoup, A. , Mortier, F. , & Cosson, J. F. (2005). A spatial statistical model for landscape genetics. Genetics, 170, 1261–1280. 15520263 10.1534/genetics.104.033803 PMC1451194

[eva12622-bib-0046] Guindon, S. , Guo, H. , & Welch, D. (2016). Demographic inference under the coalescent in a spatial continuum. Theoretical Population Biology, 111, 43–50. 27184386 10.1016/j.tpb.2016.05.002

[eva12622-bib-0047] Hand, B. K. , Cushman, S. , Landguth, E. L. , & Lucotch, J. (2014). Assessing multi‐taxa sensitivity to the human footprint, habitat fragmentation and loss by exploring alternative scenarios of dispersal ability and population size: A simulation approach. Biodiversity Conservation, 23, 2761–2779.

[eva12622-bib-0048] Hey, J. , & Machado, C. A. (2003). The study of structured populations—new hope for a difficult and divided science. Nature Reviews Genetics, 4, 535–543. 10.1038/nrg1112 12838345

[eva12622-bib-0049] Hoffmann, A. A. , & Willi, Y. (2008). Detecting genetic responses to environmental change. Nature Reviews Genetics, 9, 421–432. 10.1038/nrg2339 18463665

[eva12622-bib-0050] Holderegger, R. , & Wagner, H. H. (2008). Landscape genetics. BioScience, 58, 199–207.

[eva12622-bib-0051] Hudson, R. (1990). Gene genealogies and the coalescent process. Oxford Survey of Evolutionary Biology, 7, 1–44.

[eva12622-bib-0052] Jay, F. , Durand, E. Y. , François, O. , & Blum, M. G. B. (2015). POPS: A software for prediction of population genetic structure using latent regression models. Journal of Statistical Software, 68(9), 10.18637/jss.v068.i09

[eva12622-bib-0053] Jenkins, P. A. , Fearnhead, P. , & Song, Y. S. (2015). Tractable diffusion and coalescent processes for weakly correlated loci. Electronic Journal of Probability, 20, 1–26. 10.1214/ejp.v20-3564 PMC4929886 27375350

[eva12622-bib-0054] Joseph, T. A. , Hickerson, M. J. , & Alvarado‐Serrano, D. F. (2016). Demographic inference under a spatially continuous coalescent model. Heredity, 117, 94–99. 27118157 10.1038/hdy.2016.28 PMC4949727

[eva12622-bib-0055] Kardos, M. , Taylor, H. R. , Ellegren, H. , Luikart, G. , & Allendorf, F. W. (2016). Genomics advances the study of inbreeding depression in the wild. Evolutionary Applications, 9, 1205–1218. 27877200 10.1111/eva.12414 PMC5108213

[eva12622-bib-0056] Kelleher, J. , Barton, N. H. , & Etheridge, A. M. (2013). Coalescent simulation in continuous space. Bioinformatics, 29, 955–956. 23391497 10.1093/bioinformatics/btt067

[eva12622-bib-0057] Kelleher, J. , Etheridge, A. M. , & Barton, N. H. (2014). Coalescent simulation in continuous space: Algorithms for large neighbourhood size. Theoretical Population Biology, 95, 13–23. 24910324 10.1016/j.tpb.2014.05.001

[eva12622-bib-0058] Kelleher, J. , Etheridge, A. M. , & McVean, G. (2016). Efficient coalescent simulation and genealogical analysis for large sample sizes. PLoS Computational Biology, 12(5), e1004842 10.1371/journal.pcbi.1004842 27145223 PMC4856371

[eva12622-bib-0059] Kierepka, E. M. , & Latch, E. K. (2015). Performance of partial statistics in individual‐based landscape genetics. Molecular Ecology Resources, 15, 512–525. 25230016 10.1111/1755-0998.12332

[eva12622-bib-0060] Kimura, M. (1980). A simple method for estimating evolutionary rates of base substitutions through comparative studies of nucleotide sequences. Journal of Molecular Evolution, 16, 111–120. 7463489 10.1007/BF01731581

[eva12622-bib-0061] Kimura, M. , & Weiss, G. H. (1964). The stepping stone model of population structure and the decrease of genetic correlation with distance. Genetics, 49, 561–576. 17248204 10.1093/genetics/49.4.561 PMC1210594

[eva12622-bib-0062] Kingman, J. F. C. (1982). The coalescent. Stochastic Processes and their Applications, 13, 235–248.

[eva12622-bib-0063] Kuhner, M. K. (2008). Coalescent genealogy samplers: Windows into population history. Trends in Ecology and Evolution, 24, 86–93. 19101058 10.1016/j.tree.2008.09.007 PMC4714702

[eva12622-bib-0064] Kumar, V. , Hallström, B. M. , & Janke, A. (2013). Coalescent‐based genome analyses resolve the early branches of the euarchontoglires. PLoS ONE, 8(4), e60019 10.1371/journal.pone.0060019 23560065 PMC3613385

[eva12622-bib-0065] Laikre, L. , Allendorf, F. W. , Aroner, L. C. , Baker, C. S. , Gregovich, D. P. , Hansen, M. M. , … Waples, R. S. (2009). Neglect of genetic diversity in implementation of the Convention on Biological Diversity. Conservation Biology, 24, 86–88. 20028412 10.1111/j.1523-1739.2009.01425.x

[eva12622-bib-0066] Lankau, R. , Jørgensen, P. S. , Harris, D. J. , & Sih, A. (2011). Incorporating evolutionary principles into environmental management and policy. Evolutionary Applications, 4, 315–325. 25567975 10.1111/j.1752-4571.2010.00171.x PMC3352553

[eva12622-bib-0067] Latch, E. K. , Dharmarajan, G. , Glaubitz, J. C. , & Rhodes, O. E. Jr (2006). Relative performance of Bayesian clustering software for inferring population substructure and individual assignment at low levels of population differentiation. Conservation Genetics, 7, 295–302.

[eva12622-bib-0068] Leblois, R. , Pudlo, P. , Néron, J. , Bertaux, F. , Beeravolu, C. R. , Vitalis, R. , & Rousset, F. (2014). Maximum‐likelihood inference of population size contractions from microsatellite data. Molecular Biology and Evolution, 31, 2805–2823. 25016583 10.1093/molbev/msu212

[eva12622-bib-0069] Liu, L. , Xi, Z. , Wu, S. , Davis, C. C. , & Edwards, S. V. (2015). Estimating phylogenetic trees from genome‐scale data. Annals of the New York Academy of Sciences, 1360, 36–53. 25873435 10.1111/nyas.12747

[eva12622-bib-0070] Lloyd, M. W. , Campbell, L. , & Neel, M. C. (2013). The power to detect recent fragmentation events using genetic differentiation methods. PLoS ONE, 8(5), 63981. 10.1371/journal.pone.0063981 PMC3660580 23704965

[eva12622-bib-0071] Manel, S. , Schwartz, M. K. , Luikart, G. , & Taberlet, P. (2003). Landscape genetics: Combining landscape ecology and population genetics. Trends in Ecology and Evolution, 18, 189–197.

[eva12622-bib-0072] Mazet, O. , Rodrguez, W. , & Chikhi, L. (2015). Demographic inference using genetic data from a single individual: Separating population size variation from population structure. Theoretical Population Biology, 104, 46–58. 26120083 10.1016/j.tpb.2015.06.003

[eva12622-bib-0073] Mazet, O. , Rodríguez, W. , Grusea, S. , Boitard, S. , & Chikhi, L. (2016). On the importance of being structured: Instantaneous coalescence rates and human evolution—lessons for ancestral population size inference? Heredity, 116, 362–371. 26647653 10.1038/hdy.2015.104 PMC4806692

[eva12622-bib-0074] McRae, B. H. (2006). Isolation by resistance. Evolution, 60, 1551–1561. 17017056

[eva12622-bib-0075] Mijangos, J. L. , Pacioni, C. , Spencer, P. B. S. , & Craig, M. D. (2015). Contribution of genetics to ecological restoration. Molecular Ecology, 24, 22–37. 25377524 10.1111/mec.12995

[eva12622-bib-0076] Millete, K. L. , & Keyghobadi, N. (2015). The relative influence of habitat amount and configuration on genetic structure across multiple spatial scales. Ecology and Evolution, 5, 73–86. 25628865 10.1002/ece3.1325 PMC4298435

[eva12622-bib-0077] Milligan, B. G. (2017). Probabilistic graph models for landscape genetics. PeerJ Preprints, 5, e2225v5 10.7287/peerj.preprints.2225v5

[eva12622-bib-0078] Milligan, B. G. , Leebens‐Mack, J. , & Strand, A. E. (1994). Conservation genetics: Beyond the maintenance of marker diversity. Molecular Ecology, 3, 423–435.

[eva12622-bib-0079] Montano, V. (2016). Coalescent inferences in conservation genetics: Should the exception become the rule? Biology Letters, 12(6), 20160211 10.1098/rsbl.2016.0211 27330172 PMC4938050

[eva12622-bib-0080] Moran, P. A. P. (1958). Random processes in genetics. Mathematical Proceedings of the Cambridge Philosophical Society, 54, 60–71.

[eva12622-bib-0081] Morin, P. A. , Martien, K. K. , Archer, F. I. , Cipriano, F. , Steel, D. , Jackson, J. , & Taylor, B. L. (2010). Applied conservation genetics and the need for quality control and reporting of genetic data used in fisheries and wildlife management. Journal of Heredity, 101, 1–10. 19959596 10.1093/jhered/esp107

[eva12622-bib-0082] Murphy, M. , Dyer, R. , & Cushman, S. A. (2016). Graph theory and network models in landscape genetics In Balkenhol N., Cushman S. A., Storfer A. T. & Waits L. P. (Eds.), Landscape genetics: Concepts, methods, applications (Chap. 10, pp. 165–179). Hoboken, New Jersey: Wiley Blackwell.

[eva12622-bib-0083] Musiani, M. , Leonard, J. A. , Cluff, H. D. , Gates, C. C. , Mariani, S. , Paquet, P. C. , … Wayne, R. K. (2007). Differentiation of tundra/taiga and boreal coniferous forest wolves: Genetics, coat colour and association with migratory caribou. Molecular Ecology, 16, 4149–4170. 10.1111/j.1365-294X.2007.03458.x 17725575

[eva12622-bib-0084] Narum, S. R. , Banks, M. , Beacham, T. D. , Bellinger, M. R. , Campbell, M. R. , Dekoning, J. , … Garza, J. C. (2008). Differentiating salmon populations at broad and fine geographical scales with microsatellites and single nucleotide polymorphisms. Molecular Ecology, 17, 3464–3477. 10.1111/j.1365-294X.2008.03851.x 19160476

[eva12622-bib-0085] Neel, M. C. , McKelvey, K. , Ryman, N. , Lloyd, M. W. , Short Bull, R. , Allendorf, F. W. , … Waples, R. S. (2013). Estimation of effective population size in continuously distributed populations: There goes the neighborhood. Heredity, 111, 189–199. 23652561 10.1038/hdy.2013.37 PMC3746818

[eva12622-bib-0086] Neigel, J. E. (2002). Is *F* _ST_ obsolete? Conservation Genetics, 3, 167–173.

[eva12622-bib-0087] Notohara, M. (1990). The coalescent and the genealogical process in geographically structured populations. Journal of Mathematical Biology, 29, 59–75. 2277236 10.1007/BF00173909

[eva12622-bib-0088] Ortego, J. , Garca‐Navas, V. , Noguerales, V. , & Cordero, P. J. (2015). Discordant patterns of genetic and phenotypic differentiation in five grasshopper species codistributed across a microreserve network. Molecular Ecology, 24, 5796–5812. 26475782 10.1111/mec.13426

[eva12622-bib-0089] Ovenden, J. R. , Berry, O. , Welch, D. J. , Buckworth, R. C. , & Dichmont, C. M. (2015). Ocean's eleven: A critical evaluation of the role of population, evolutionary and molecular genetics in the management of wild fisheries. Fish and Fisheries, 16, 125–159.

[eva12622-bib-0090] Oyler‐McCance, S. J. , Fedy, B. C. , & Landguth, E. L. (2013). Sample design effects in landscape genetics. Conservation Genetics, 14, 275–285.

[eva12622-bib-0091] Paz‐Vinas, I. , Comte, L. , Chevalier, M. , Dubut, V. , Veyssiere, C. , Grenouillet, G. , … Blanchet, S. (2013). Combining genetic and demographic data for prioritizing conservation actions: Insights from a threatened fish species. Ecology and Evolution, 3, 2696–2710. 24567833 10.1002/ece3.645 PMC3930054

[eva12622-bib-0092] Peery, M. Z. , Kirby, R. , Reid, B. N. , Stoelting, R. , Doucet‐Bëer, E. , Robinson, S. , … Palsbøll, P. J. (2012). Reliability of genetic bottleneck tests for detecting recent population declines. Molecular Ecology, 21, 3403–3418. 22646281 10.1111/j.1365-294X.2012.05635.x

[eva12622-bib-0093] Pertoldi, C. , Bijlsma, R. , & Loeschcke, V. (2007). Conservation genetics in a globally changing environment: Present problems, paradoxes and future challenges. Biodiversity Conservation, 16, 4147–4163.

[eva12622-bib-0094] Pierson, J. C. , Coates, D. J. , Oostermeijer, J. G. B. , Beissinger, S. R. , Bragg, J. G. , Sunnucks, P. , & Young, A. G. (2016). Genetic factors in threatened species recovery plans on three continents. Frontiers of Ecology and the Environment, 14, 433–440.

[eva12622-bib-0095] Pritchard, J. K. , Stephens, M. , & Donnelly, P. (2000). Inference of population structure using multilocus genotype data. Genetics, 155, 945–959. 10835412 10.1093/genetics/155.2.945 PMC1461096

[eva12622-bib-0096] Rodrguez‐Trelles, F. , & Rodrguez, M. Á. (2010). Measuring evolutionary responses to global warming: Cautionary lessons from Drosophila. Insect Conservation and Diversity, 3, 44–50.

[eva12622-bib-0097] Rousset, F. (1997). Genetic differentiation and estimation of gene flow from *F*‐statistics under isolation by distance. Genetics, 145, 1219–1228. 9093870 10.1093/genetics/145.4.1219 PMC1207888

[eva12622-bib-0098] Samarasin, P. , Shuter, B. J. , Wright, S. I. , & Rodd, F. H. (2017). The problem of estimating recent genetic connectivity in a changing world. Conservation Biology, 31, 126–135. 27235331 10.1111/cobi.12765

[eva12622-bib-0099] Schwartz, M. K. , Luikart, G. , & Waples, R. S. (2007). Genetic monitoring as a promising tool for conservation and management. Trends in Ecology and Evolution, 22, 25–33. 16962204 10.1016/j.tree.2006.08.009

[eva12622-bib-0100] Schwartz, M. K. , & McKelvey, K. S. (2009). Why sampling scheme matters: The effect of sampling scheme on landscape genetic results. Conservation Genetics, 10, 441–452.

[eva12622-bib-0101] Segelbacher, G. , Cushman, S. A. , Epperson, B. K. , Fortin, M.‐J. , Francois, O. , Hardy, O. J. , & Manel, S. (2010). Applications of landscape genetics in conservation biology: Concepts and challenges. Conservation Genetics, 1, 375–385.

[eva12622-bib-0102] Serrouya, R. , Paetkau, D. , McLellan, S. , Boutin, B. N. , Jenkins, D. , & Campbell, M. (2012). Population size and major valleys explain microsatellite variation better than taxonomic units for caribou in western Canada. Molecular Ecology, 21, 2588–2601. 22502637 10.1111/j.1365-294X.2012.05570.x

[eva12622-bib-0103] Slatkin, M. (1985). Gene flow in natural populations. Annual Review of Ecology and Systematics, 16, 393–430.

[eva12622-bib-0104] Slatkin, M. (1993). Isolation by distance in equilibrium and non‐equilibrium populations. Evolution, 47, 264–279. 28568097 10.1111/j.1558-5646.1993.tb01215.x

[eva12622-bib-0105] Smith, C. T. , Antonovich, A. , Templin, W. D. , Elfstrom, C. M. , Naurm, S. R. , & Seeb, L. W. (2007). Impacts of marker class bias relative to locus‐specific variability on population inferences in Chinook salmon: A comparison of single‐nucleotide polymorphisms with short tandem repeats and allozymes. Transactions of the American Fisheries Society, 136, 1674–1687. 10.1577/T06-227.1

[eva12622-bib-0106] Smith, C. T. , & Seeb, L. W. (2008). Number of alleles as a predictor of the relative assignment accuracy of short tandem repeat (STR) and single‐nucleotide‐polymorphism (SNP) baselines for chum salmon. Transactions of the American Fisheries Society, 137, 751–762. 10.1577/T07-104.1

[eva12622-bib-0107] Spear, S. F. , Cushman, S. A. , & McRae, B. H. (2016). Resistance surface modeling in landscape genetics In Balkenhol N., Cushman S. A., Storfer A. T. & Waits L. P. (Eds.), Landscape genetics: Concepts, methods, applications (Chap. 8, pp. 129–148). Hoboken, New Jersey: Wiley Blackwell.

[eva12622-bib-0108] Storfer, A. , Murphy, M. A. , Spear, S. F. , Holderegger, R. , & Waits, L. P. (2010). Landscape genetics: Where are we now? Molecular Ecology, 17, 3496–3514. 10.1111/j.1365-294X.2010.04691.x 20723061

[eva12622-bib-0109] Strand, A. E. , Milligan, B. G. , & Pruitt, C. M. (1996). Are populations islands? Analysis of chloroplast DNA variation in *Aquilegia* . Evolution, 50, 1822–1829. 28565584 10.1111/j.1558-5646.1996.tb03568.x

[eva12622-bib-0110] Tallmon, D. A. , Gregovich, D. , Waples, R. S. , Baker, C. S. , Jackson, J. , Taylor, B. L. , … Schwartz, M. K. (2010). When are genetic methods useful for estimating contemporary abundance and detecting population trends? Molecular Ecology Resources, 10, 684–692. 21565073 10.1111/j.1755-0998.2010.02831.x

[eva12622-bib-0111] Tellier, A. , & Lemaire, C. (2014). Coalescence 2.0: A multiple branching of recent theoretical developments and their applications. Molecular Ecology, 23, 2637–2652. 24750385 10.1111/mec.12755

[eva12622-bib-0112] Véber, A. , & Wakolbinger, A. (2015). The spatial Lambda‐Fleming‐Viot process: An event‐based construction and a lookdown representation. Annales de l'Institut Henri Poincaré, Probabilités et Statistiques, 51, 570–598.

[eva12622-bib-0113] Waits, L. P. , & Storfer, A. (2016). Basics of population genetics: Quantifying neutral and adaptive genetic variation for landscape genetic studies In Balkenhol N., Cushman S. A., Storfer A. T. & Waits L. P. (Eds.), Landscape genetics: Concepts, methods, applications (Chap. 3, pp. 35–57). Hoboken, New Jersey: Wiley Blackwell.

[eva12622-bib-0114] Waples, R. S. (1998). Separating the wheat from the chaff: Patterns of genetic differentiation in high gene flow species. Journal of Heredity, 89, 438–450.

[eva12622-bib-0115] Waples, R. S. , Punt, A. E. , & Cope, J. M. (2008). Integrating genetic data into management of marine resources: How can we do it better? Fish and Fisheries, 9, 423–449.

[eva12622-bib-0116] Wasserman, T. N. , Cushman, S. A. , Schwartz, M. K. , & Wallin, D. O. (2010). Spatial scaling and multi‐model inference in landscape genetics: *Martes Americana* in northern Idaho. Landscape Ecology, 25, 1601–1612.

[eva12622-bib-0117] Weiss, G. H. , & Kimura, M. (1965). A mathematical analysis of the stepping stone model of genetic correlation. Journal of Applied Probability, 2, 129–149.

[eva12622-bib-0118] Whitlock, M. C. , & McCauley, D. E. (1999). Indirect measures of gene flow and migration: *F* _ST_ ≠ 1/(4*Nm* + 1). Heredity, 82, 117–125. 10098262 10.1038/sj.hdy.6884960

[eva12622-bib-0119] Wright, S. (1931). Evolution in Mendelian populations. Genetics, 16, 97–159. 17246615 10.1093/genetics/16.2.97 PMC1201091

[eva12622-bib-0120] Wright, S. (1943). Isolation by distance. Genetics, 28, 114–138. 17247074 10.1093/genetics/28.2.114 PMC1209196

[eva12622-bib-0121] Wright, S. (1946). Isolation by distance under diverse systems of mating. Genetics, 31, 39–59. 21009706 10.1093/genetics/31.1.39 PMC1209315

[eva12622-bib-0122] Wright, S. (1965). The interpretation of population structure by *F*‐statistics with special regard to systems of mating. Evolution, 19, 395–420.

